# Correction to: The effect of methanol fixation on single-cell RNA sequencing data

**DOI:** 10.1186/s12864-021-07806-9

**Published:** 2021-07-19

**Authors:** Xinlei Wang, Lei Yu, Angela Ruohao Wu

**Affiliations:** 1 Division of Life Science, Hong Kong University of Science and Technology, Clear Water Bay, Hong Kong SAR, China; 2 Department of Chemical and Biological Engineering, Hong Kong University of Science and Technology, Clear Water Bay, Hong Kong SAR, China; 3 Hong Kong Branch of Guangdong Southern Marine Science and Engineering Laboratory (Guangzhou), Hong Kong University of Science and Technology, Clear Water Bay, Hong Kong SAR, China

**Correction to: BMC Genomics 22, 420 (2021)**

**https://doi.org/10.1186/s12864-021-07744-6**

Following publication of the original article [1], it was reported that Fig. 1 and Fig. 3 contained errors.

Figure 1C text labels were resized for readability, and Fig. 3C contained an error in the y-axis.

The correct figures and captions are given in this Correction article and the original article has been updated.

**Fig. 1:**
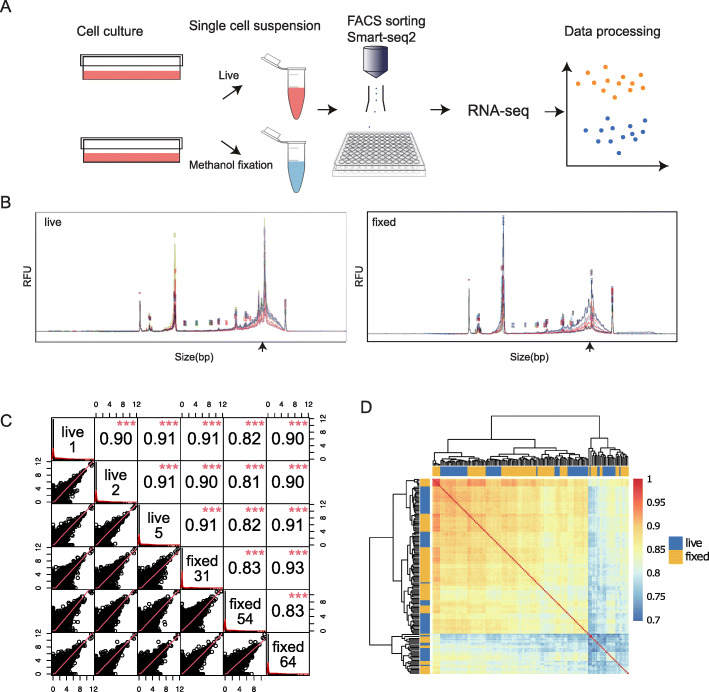
Basic evaluation of fixation effect on sequencing data. (**A**) Workflow and experimental scheme (**B**) Size distributions of cDNA libraries. Traces from single-cell libraries were merged to obtain a general pattern for live (left) and fixed (right) samples. Although the intensity of the ~ 1500 bp peak (pointed by arrow on size axis) is diminished in fixed cells, there is no visible degradation. (**C**) Correlation matrix showing the transcriptome similarity of cells randomly chosen from live and fixed samples. The upper triangle of the matrix shows the Pearson correlation coefficient and the bottom triangle visualized correlation trend. Correlations are consistently high for both inter- and intra-treatment comparisons of live vs. fixed. There is no obvious bias revealed by measuring correlation between single-cell transcriptomes for all pairwise comparisons. (**D**) Correlation factors of all single cells were calculated pairwise and clustered by Euclidean distance. Correlations are consistently high for both inter- and intra-treatment comparisons of live vs. fixed (R2 > 0.7). The mixed annotation bar indicates the transcriptome similarities do not distinguish cell treatments during sample preparation

**Fig. 3:**
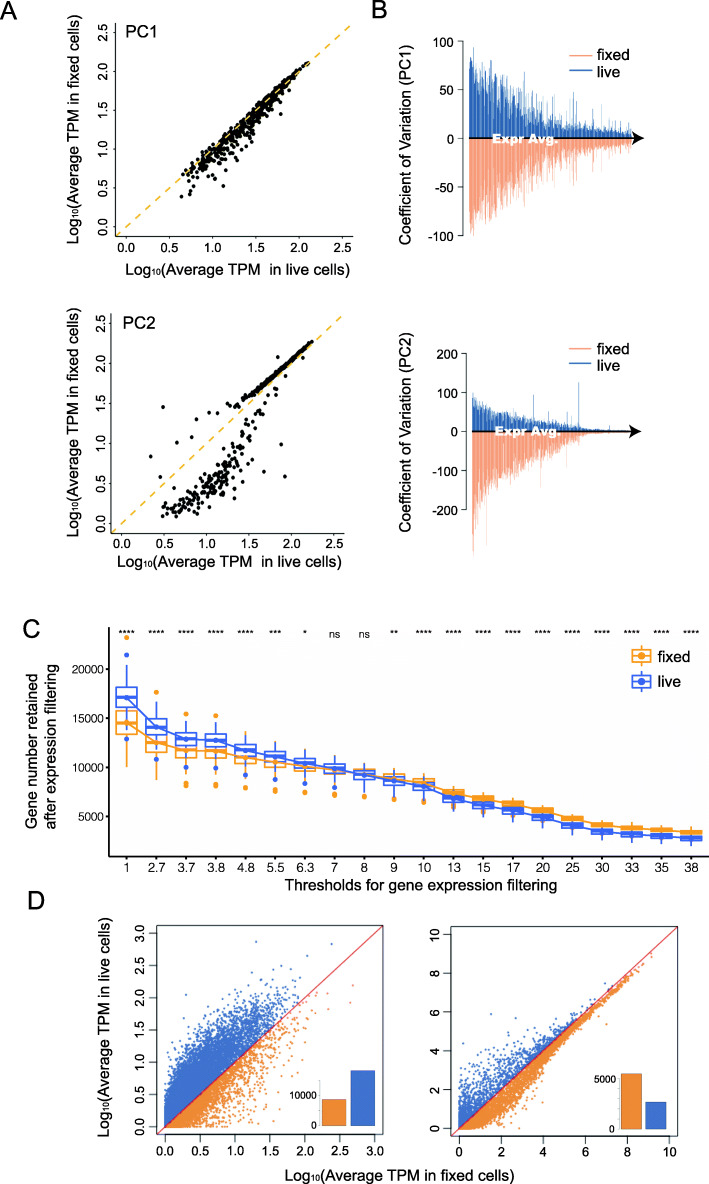
Differences in statistical features of genes with the top contribution in driving variation between live and fixed cells. (**A**) Comparison of relative expression of 500 genes with the top contribution in PC1 and PC2 between live and fixed cells. Expression of PC1 genes correlated well while in PC2 the trend was incoherent for genes with different expressions level, which indicates genes heavily loaded in PC2 may be responsible for the separation between two groups of cells. (**B**) Comparison of expression variation of genes with top contribution from PC1 and PC2. In the top panels of Fig. 3B, we take genes that are heavily loaded in PC1 respect for live cells and fixed cells. Then, we computed the coefficient of variation (CV) of each gene across all cells. The CVs for each gene are then plotted against that gene’s mean expression level, separately for live (blue) and fixed (orange) cells. Genes with the top contribution in PC2 holds much higher variation compared with PC1 genes. (**C**) Comparison of gene detection number after expression filtering. A series of thresholds were set up for different sensitivity requirements. The detection number in fixed cells gradually surpass live cells once the threshold increased (nsP > 0.05, *P < 0.05,**P < 0.01,****P < 0.0001). (**D**) Relative abundances of genes with low (< 5 TPM) or high (> 30 TPM) expression, the inset bar charts compare the quantities of genes which have higher expression in either live (blue) and fixed (orange) cells. For low expression genes, they are generally more abundant in live cells. Genes with higher expression are more abundant in fixed cells
