# Supplementary material for: The effect of methanol fixation on single-cell RNA sequencing data
Source: BMC Genomics. 2021 Jun 5;22:420. doi: 10.1186/s12864-021-07744-6 (PMC8180132; doi:10.1186/s12864-021-07744-6)
Supplement: Supplementary file 1 — Additional file 1: Supplementary Figure 1. Statistical features of genes with the top contribution for driving different PCs between live and fixed cells in HepG2. (A) Comparison of relative expression of 500 genes with the top contribution in PC1 (top) and PC2 (bottom) between live and fixed cells. (B) Comparison of expression variation of genes with top contribution from PC1 (top) and PC2 (bottom). (C) Relative abundances of genes with low (<5 TPM, left) high (>30 TPM, right) expression, the inset bar charts compare the quantities of genes that have higher expression in either live (blue) and fixed (orange) cells. (D) Comparison of gene detection number after expression filtering. Supplementary Figure 2. Molecular features of transcripts separating PC1 and PC2 in HepG2. (A) Plots of GC content and corresponding rank for the whole transcriptome. Highlighted events are those with top contributions in PC1 (left) and PC2 (right). (B) Plots of length and corresponding rank for the whole transcriptome. Highlighted events are those with top contributions in PC1 (left) and PC2 (right). (C) Comparisons of length (left) and GC content (right) of transcripts with top contributions in PC1 and PC2. P-values show differences between live and fixed groups are both significant. (D) Comparison of transcripts detection number. Groups are separated and arranged by increasing length. The number of transcript detection varies as length changes. Statistical significance p-values are determined by t-test and indicated with asterisks (ns P>0.05, *P<0.05, ****P<0.0001). Supplementary Figure 3. Comparison of mapping features between live and fixed cells in HepG2. (A) The mapping ratios for each transcript were compared using coverage integrity correlation. Transcripts with top or bottom 10% ranking in length and GC content are highlighted in each correlation plot. (B) Visualization of the ratio of live/fixed mapping integrity. Transcripts are sorted and grouped by length and GC content; ea [file 12864_2021_7744_MOESM1_ESM.docx]

**Supplementary information**

**Effect of methanol fixation on single-cell RNA sequencing data**

Authors:

Xinlei WANG, Lei YU, Angela WU.

**Supplementary Figure 1.** Statistical features of genes with the top contribution for driving different PCs between live and fixed cells in HepG2.

**(A)** Comparison of relative expression of 500 genes with the top contribution in PC1(top) and PC2(bottom) between live and fixed cells.

(**B**) Comparison of expression variation of genes with top contribution from PC1(top) and PC2(bottom)

(**C**) Relative abundances of genes with low (<5 TPM, left) high (>30 TPM, right) expression, the inset bar charts compare the quantities of genes that have higher expression in either live(blue) and fixed(orange) cells.

(**D**) Comparison of gene detection number after expression filtering.

**Supplementary Figure 2.** Molecular features of transcripts separating PC1 and PC2 in HepG2.

(**A**) Plots of GC content and corresponding rank for the whole transcriptome. Highlighted events are those with top contributions in PC1 (left) and PC2 (right).

(**B**) Plots of length and corresponding rank for the whole transcriptome. Highlighted events are those with top contributions in PC1 (left) and PC2 (right).

(**C**) Comparisons of length (left) and GC content (right) of transcripts with top contributions in PC1 and PC2. P-values show differences between live and fixed groups are both significant.

(**D**) Comparison of transcripts detection number. Groups are separated and arranged by increasing length. The number of transcript detection varies as length changes. Statistical significance p-values are determined by t-test and indicated with asterisks (nsP>0.05, *P<0.05, ****P<0.0001).

**Supplementary Figure 3.** Comparison of mapping features between live and fixed cells in HepG2.

**(A)** The mapping ratios for each transcript were compared using coverage integrity correlation. Transcripts with top or bottom 10% ranking in length and GC content are highlighted in each correlation plot.

**(B)** Visualization of the ratio of live/fixed mapping integrity. Transcripts are sorted and grouped by length and GC content; each unit represents an average ratio for those transcripts in one group.

**Supplementary Figure 4.** Transcripts with longer length and higher GC content separate live and fixed cells (done using HepG2).

(**A**) PCA performed using different transcripts sets. In each plot, transcripts are selected based on length and GC content thresholds.

(**B**) PC1 loadings of cells in PCAs performed with different sets of transcripts.

(**C**) With PCAs performed with increasing length and GC content thresholds, corresponding length or GC content statistic of the top 500 transcripts from PC1s was plotted.

**Supplementary Figure 5.** PCA using HepG2 data generated by mapping raw reads to 3’end (A) and 5’end (B) of transcripts.

HCT-116

HepG2

**Supplementary Figure 6.** Expression correlation of transcripts with different lengths. Transcripts are equally grouped to 16 according to the length. IDs in each plot represent the transcripts group included in that plot. With the increase of the ID number, the average length of the transcript group also increased. Both results of HCT-116(top) and HepG2(bottom) are shown.

HCT-116

HepG2

**Supplementary Figure 7.** Comparison of mapping coverage between live and fixed cells. Transcripts are equally grouped to 10 according to the length. IDs in each plot represent the transcripts group included in that plot. With the increase of the ID number, the average length of that transcript group also increased. Both results of HCT-116 (top) and HepG2 (bottom) are shown.

**Supplementary Figure 8.** Basic evaluation of methanol fixation protocol

1. Fastqc result of data of both live and fixed cells.
2. Ratio of mapped reads of live and fixed cells.
3. Percentage of GC in raw sequence reads from live and fixed data.
4. FACS gating for single cell sorting.
5. Cell counter images of cells during sample processing steps. Left panel shows state of cell suspension upon digestion and fixation. Middle panel shows the look of cells filtered by cell strainers. Right panel shows images of cells sorted by FACS.

**
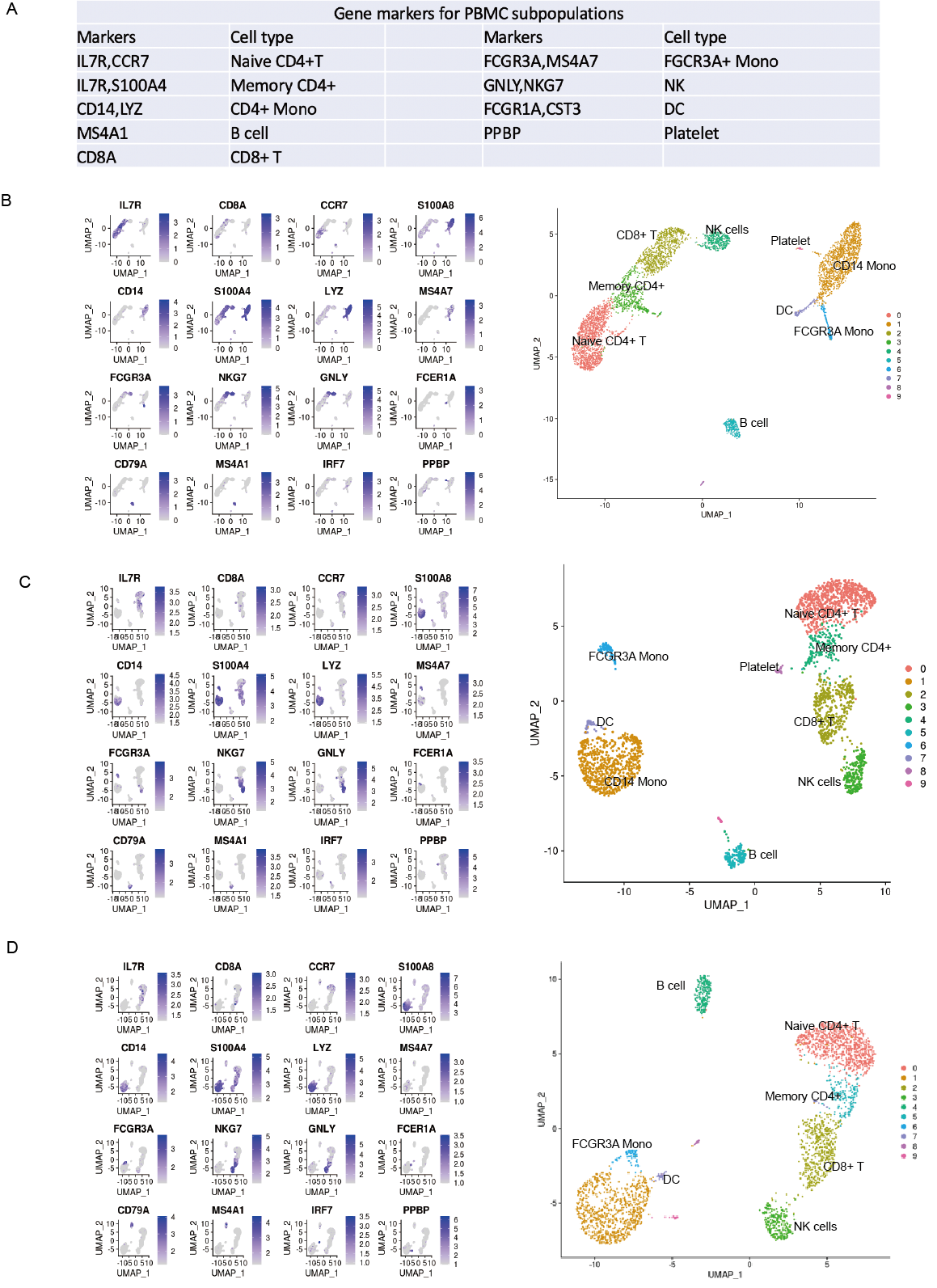
**

**Supplementary Figures 9.** PBMC analysis validates the feasibility of methanol fixation in tissue samples.

1. Gene markers of PBMC subtypes learned from Seurat tutorial (<https://satijalab.org/seurat/archive/v3.0/immune_alignment.html>).

(B-D) Left panels show UMAP of PBMC data generated from different sample conditions with gene markers highlighted. Right panels show the UMAP of PBMC data from different conditions with cell types annotated.

**Supplementary Figure 10.** Methanol fixation does not cause cell-type specific drop-out in single cell RNA-seq data.

1. UMAP plotted by data integrated from all sample conditions with cell type annotated.
2. UMAP plotted by integrated data with gene markers highlighted.
3. UMAP plotted using integrated data and split according to sample conditions.
4. Frequency of each PBMC subtype in data sets generated from each condition.
